# Crystalline and oxide phases revealed and formed on InSb(111)B

**DOI:** 10.1038/s41598-018-32723-5

**Published:** 2018-09-26

**Authors:** Jaakko Mäkelä, Zahra Sadat Jahanshah Rad, Juha-Pekka Lehtiö, Mikhail Kuzmin, Marko P. J. Punkkinen, Pekka Laukkanen, Kalevi Kokko

**Affiliations:** 1 University of Turku, Department of Physics and Astronomy, FI-20014 Turku, Finland; 2 Comptek Solutions Ltd., Voimakatu 14, FI-20520 Turku, Finland; 3 Ioffe Physical-Technical Institute, Russian Academy of Sciences, St. Petersburg, 194021 Russian Federation

## Abstract

Oxidation treatment creating a well-ordered crystalline structure has been shown to provide a major improvement for III–V semiconductor/oxide interfaces in electronics. We present this treatment’s effects on InSb(111)B surface and its electronic properties with scanning tunneling microscopy and spectroscopy. Possibility to oxidize (111)B surface with parameters similar to the ones used for (100) surface is found, indicating a generality of the crystalline oxidation among different crystal planes, crucial for utilization in nanotechnology. The outcome is strongly dependent on surface conditions and remarkably, the (111) plane can oxidize without changes in surface lattice symmetry, or alternatively, resulting in a complex, semicommensurate quasicrystal-like structure. The findings are of major significance for passivation via oxide termination for nano-structured III–V/oxide devices containing several crystal plane surfaces. As a proof-of-principle, we present a procedure where InSb(111)B surface is cleaned by simple HCl-etching, transferred via air, and post-annealed and oxidized in ultrahigh vacuum.

## Introduction

InSb crystals have been increasingly investigated in order to develop low band gap (~0.2 eV) and high electron mobility (~80 000 cm^2^ V^−1^ s^−1^) demanding electronics devices, but also various prospective future applications. For example, InSb is utilized nowadays in infrared detectors and is very potential for high-speed transistors operating at low voltages^1–6^ and other ultra thin device applications^7,8^, and very recently, e.g., as building blocks of quantum computers^9^ and THz transport waveguides^10^. The common challenge in developing these various InSb-based devices is how the surface or interface properties of InSb crystals can be modified in controlled manner. The surface properties of InSb increasingly affect the operation of devices because the size of individual components is systematically becoming smaller. One of the major challenges with InSb surfaces (and with semiconductor surfaces in general) is their strong and uncontrolled reactivity with environment (e.g., in air and growth conditions of many thin films). In particular, InSb surfaces become oxidized very spontaneously. Thus, it is very difficult in practice to avoid the formation of natively oxidized surface on InSb crystals during the manufacturing of the devices. Furthermore, because such native oxide films usually lack long range order and are rich in defects, including high densities of electronic defects levels at the interface, these InSb surfaces oxidized in uncontrolled manner are a performance limiting part of many devices. Indeed, controlled modifications of the oxidized surfaces have been intensively researched and developed^7,11–13^.

In contrast to the traditional target of avoiding InSb native oxide formation, we present here an opposite approach where InSb surfaces are intentionally pre-oxidized in such way that the oxidized surfaces still remain crystalline. This is the same method we have used previously for preparing crystalline oxidized III–V(100) surfaces^14^, found to reduce the amount of defect states by over an order of magnitude beneath an atomic layer deposition (ALD) grown oxide film^15^ and thus enabling efficient passivation. However, the following crucial questions have previously remained unresolved: (i) is the crystalline oxidation possible for other crystal planes; in more general for various crystal faces [not only (100) planes] exposed in nanotechnology^2,7–9,12,16,17^, and (ii) how to produce clean InSb surfaces in an industrially potential way, which is necessary in order to perform the crystalline pre-oxidation in practice. Here we provide solutions to these questions using InSb(111)B as a template for investigations.

Clean InSb(111)B exhibits (3 × 3) reconstruction in In-rich or high temperature conditions and (2 × 2) reconstruction in Sb-rich or lower temperature conditions^18–20^. Also, observation of a high-temperature (3 × 1) phase has been reported at high temperature^21–23^, assumed to be caused by detachment of atomic rows beneath the reconstructed topmost layer due to the volatility of Sb^23^.

Scanning tunneling microscopy (STM) and spectroscopy (STS) are powerful tools for investigating monolayer structures and adsorption induced differences in topographic and electronic properties for atomically smooth samples. In particular, STS can provide information about prominent electronic states at or around the band gap area of the sample surface energy band and/or the pinning of Fermi-level^24,25^. Current imaging tunneling spectroscopy (CITS) is very useful in differentiating different electronic properties that possibly exist in two different lateral positions on the surface. These tools are utilized here to observe effects on InSb(111)B induced by oxidation treatments.

The generality of the crystalline oxidation might remain readily hidden because the lattice periodicity can change surprisingly little as compared to the (100) surfaces. Therefore we also discuss and relate the behavior and properties of clean InSb(111)B to our observations. Furthermore, in some circumstances, oxidation can induce more drastic changes, resulting in a quasicrystal-like surface. These effects observed here will be discussed in detail.

## Results and Discussion

### Clean surface

**Figure 1:**
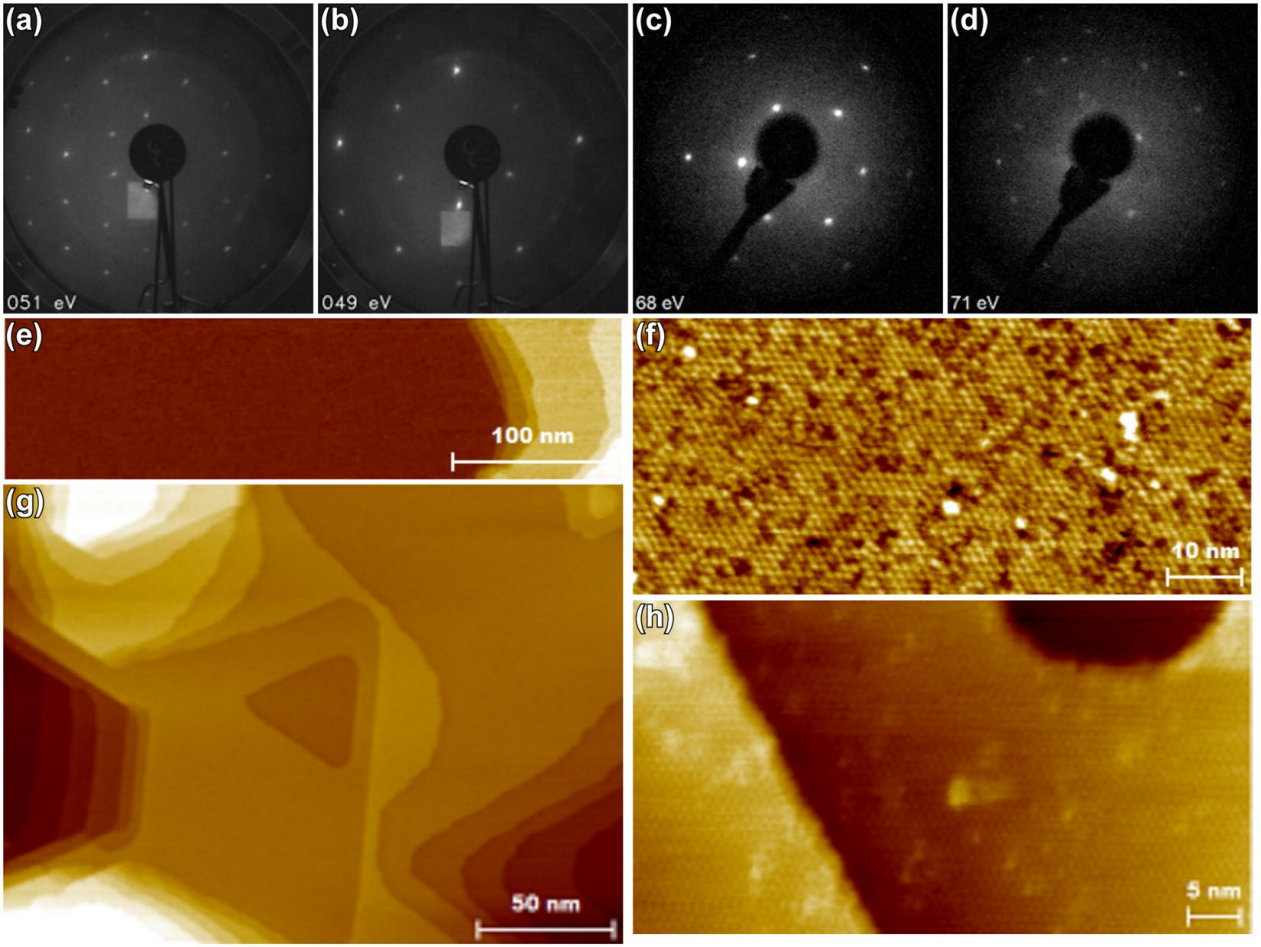
LEED images from clean (3 × 3) surface after sputter-cleaning (**a**), (2 × 2) surface after Sb-deposition, (**b**). Faint rings at about 2/3 of the radius originate from reflections at the viewport in (**a**,**b**). From the other UHV system with sample rotated 90°: after HCl + 300 °C (**c**) and HCl + H_2_ + 400 °C anneal (**d**). STM from the (3 × 3) surface in (**d**) with *I*_*t*_ = 55 pA, *V*_*g*_ = −0.88 V (**e**), *I*_*t*_ = 55 pA, *V*_*g*_ = −1.00 V (**f**). RT STM images of (2 × 2) surface after Sb deposition: large (**g**) and narrow (**h**) scale, *I*_*t*_ = 55 pA, *V*_*g*_ = −0.80 V.

Simple UHV cleaning produced a smooth InSb(111)B(3 × 3) surface, which was utilized as a template in further experiments. The In-rich surfaces resulting from sputtering are assumed to be favorable when producing the crystalline oxidation, which seems to stabilize well with In-rich (100) surfaces^14^. After deposition of Sb on the sample surface, prominent (2 × 2) reconstruction was observed with LEED without any residual traces of (3 × 3). Figure 1 shows an example of these reconstructions in (a) and (b). We were also able to produce both reconstructions by cleaning the sample in atmosphere with HCl treatment in air and subsequent anneal in either UHV or H_2_ ambient. (2 × 2) was produced by post-annealing of 300 °C and (3 × 3) using 360 °C or higher temperatures as seen in Fig. 1(c,d). STM images with distinct atomic smoothness and (3 × 3) features are observable in Fig. 1(e,f). To confirm optimally clean starting conditions and to be able to utilize same sample for multiple experiments, mainly sputtering and annealing treatments were used to clean the samples in further experiments, but it is noteworthy that the effects which are discussed here were observed also for samples cleaned with HCl. STM images from (2 × 2)-Sb are shown in Fig. 1(g,h).

After annealing at 470 °C, still only (3 × 3) could be observed with LEED after cooling to RT. It is possible that (3 × 3) and (3 × 1) reconstructions coexist on macroscopic scale since the LEED spots from such structures would overlap. If this is the case, individual effects on oxidation from each phase cannot be strictly separated or assigned to either one on an atomic scale since we do not have possibility for STM scanning with simultaneous oxidation treatments to make local distinction between the phases. It is to be noted at this stage, that the 470 °C annealing treatment resulted in slight melting or roughening of the sample, observed as matt appearance of the surface, from one edge that might have been exposed to even higher temperature due to slight temperature gradient caused by the sample holder. The sample still showed a clear (3 × 3) reconstruction from the matt edge as also from the mirror-like edge that is seen on a fresh sample (we denote such surface henceforth “pristine”). Similar surface was observed with naked eye for samples after a number of oxidation tests, and/or prolonged higher temperature annealing or higher acceleration voltage sputtering treatments. Such areas typically showed facetted, expanded pit areas with some clustering in STM, but revealing also smooth areas in large scale as expected from (3 × 3) LEED observation. The implications of the surface that is visibly worn out or matt (henceforth “roughened”) as described earlier, has remarkable effects on the oxidation and will be discussed later.

**Figure 2:**
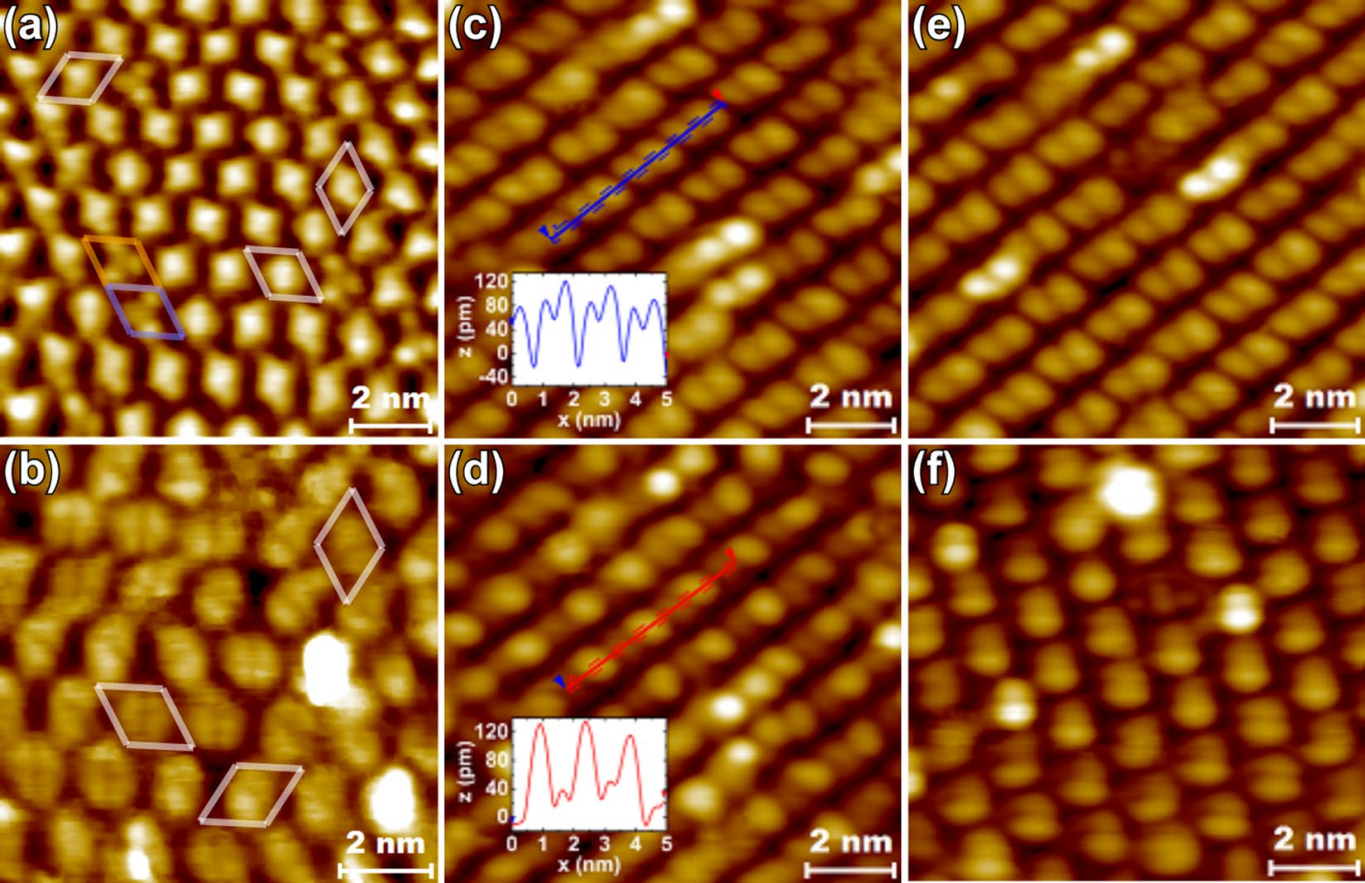
STM images at RT: (**a**) filled and (**b**) empty states with (3 × 3) sized boundaries surrounding differently oriented hexamers (different colors for different types of hexamers), and at LNT (empty states): (**c**,**d**) showing comparison between line profiles along hexamers in two scans with 6 min time interval, (**e**,**f**) showing comparison between hexamer orientations with 70 min time interval.

**Figure 3:**
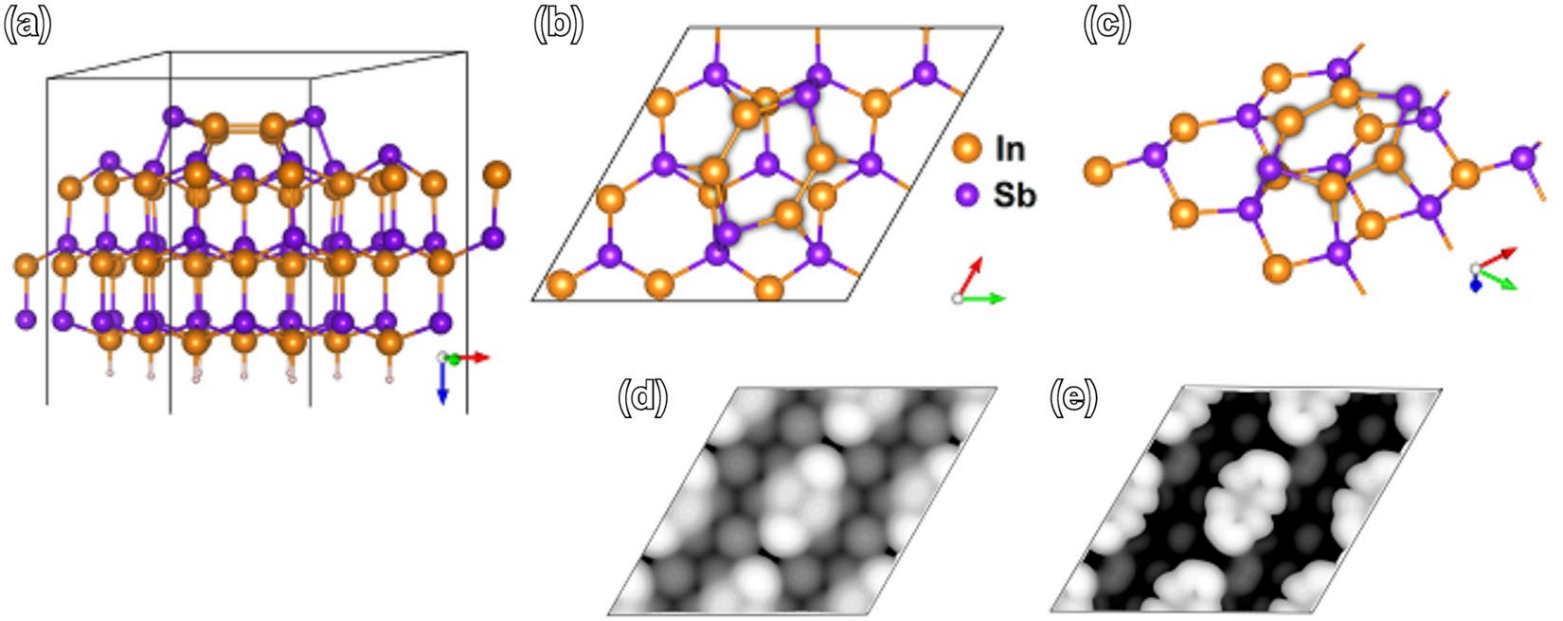
Atomic model for an In_4_Sb_2_ hexamer unit cell on InSb(111)B^20^ (**a**–**c**). VESTA 3 software^60^ has been used for visualizing the models. Lower pane contains constant current STM simulations for the atomic models of clean (3 × 3) surface as in (**a**–**c**), matching with the measured STM images in Fig. 2 for filled states, −1 V (**d**), and empty states, 1 V (**e**).

So far, no competing atomic models have been proposed in the literature for the (3 × 3) model by Wever *et al*.^20^, which is within reasonable agreement with also other experimental studies^26,27^. Figure 2(a,b) show RT STM images measured from the clean sample consistent with the earlier studies. In Fig. 2(a), three different types of cell structures (marked with differently colored borders), each having random orientations separated by rotations of 60° are observed. These features arise from hexamer rings on the surface having stoichiometry of In_4_Sb_2_ (white and blue boundaries) or In_3_Sb_3_ (orange boundary)^20^. Figure 3 shows unit cell of the regular, dominating In_4_Sb_2_ structure from our computational model based on the structure as in ref.^20^ that covers majority of the surface area. The STM simulations that closely resemble our measured data (Fig. 2) are shown in Fig. 3(d,e), and give further support for the validity of the model. In our experiments, (3 × 3) is always the starting surface, and we will use the model as the basis for our discussion.

The random orientations separated by angles of 60° of hexamer unit cells that are prone to fluctuation in RT^20^ were found to freeze in parallel orientation in LNT STM. During scanning, however, the nominally symmetrical protrusions on two sides of a hexamer are found to become tilted in height or electrical net charge and seen as the other side of the hexamer being brighter in the STM images. This effect is seen in similar proportion and on the same side of all of the hexamers in a single scan image, illustrated by Fig. 2(c,d) separated only by 6 minutes of scanning on the same area.

As shown in Fig. 3, each of the hexamer atoms has one bond with an Sb atom of the 1st atomic double layer. Thus, random fluctuation of hexamer orientation is an energetically expensive process, surprisingly enabled by RT, albeit slowly. Room temperature observations also show that there is no significant net polarization of charge in hexamers that would affect the orientation of an adjacent In_4_Sb_2_ hexamer between bond breaking and rebonding. This is particularly interesting since i) the model does not follow the electron counting rule (ECR)^28^ and has an excess electronic charge of 0.75 e per unit cell, ii) the mirror symmetry of In_4_Sb_2_ is absent in another type of hexamer, In_3_Sb_3_, on the surface and the (111) plane of zincblende InSb. Because the surface does not obey ECR, part of the dangling bonds of In (one DB per In atom) becomes occupied by electrons. It can be expected that excess electrons can move easily inside one hexamer via nearby In dangling bonds but in contrast it is not so probable that electron charge is transferred between the hexamers. Thus, it would be a natural consequence for the excess static charge to build up on one side of a fluctuating hexamer to accommodate smallest amount of repulsive Coulombic force and thus create a chain effect in which adjacent hexamers would build up similar charge on the same side and change orientation correspondingly due to repulsive forces (like van der Waals bonding). However, such an effect is observed for cleaned pristine surface only in LNT.

An analoguous situation regarding freezing of reconstruction and static charge distribution is found on Si(100)(2 × 1)^29,30^ and Ge(100)(2 × 1)^31,32^, which have a minimum energy configuration in a buckled dimer orientation in which another atom of a surface dimer has an empty dangling bond and another a filled one. Observation of (2 × 1) reconstruction arises due to fluctuation of charge between the dangling bonds in RT. Indeed, in LNT the corresponding surfaces can readily be seen as alternating orientations of buckled dimers in dimer rows, i.e., in *p*(2 × 2) or *c*(4 × 2) configurations, which have a low energy barrier between them. Similar freezing is caused by local defects due to strain effects and electrostatics and this effect can extend much beyond individual dimers as a chain effect.

We expect the freezing effect of hexamers on InSb(111)B(3 × 3) to be in some sense similar, since LNT seems to stabilize the parallel orientations. It is clear from our results that electrostatics has a significant effect on the STM observations through disruption of the mirror symmetricity of hexamers as charge build up, possibly in a similar nature as on Ge(100), on which reversible, local *p*(2 × 2) to *c*(4 × 2) transition is possible through charge injection from STM tip^31^. However, the overall mechanism is in any case markedly different in our case since the hexamer charge build up tilting is much less stable and we have not found tunneling conditions that would correspond to a given orientation. Instead, chain effect stabilization or electrostatic interaction between adjacent dimers or polarized defects is found relatively strong, since a whole scanning area is prone to shift in orientation (see Fig. 2(e,f)), possibly due to gentle tip crash or other instability, or adventitious impurity adsorption. Thus, lattice strain is suggested to have a less significant effect in the case of InSb(111)B(3 × 3) since it would be expected to have more of a stabilizing effect locally.

### Oxidation-induced changes

**Figure 4:**
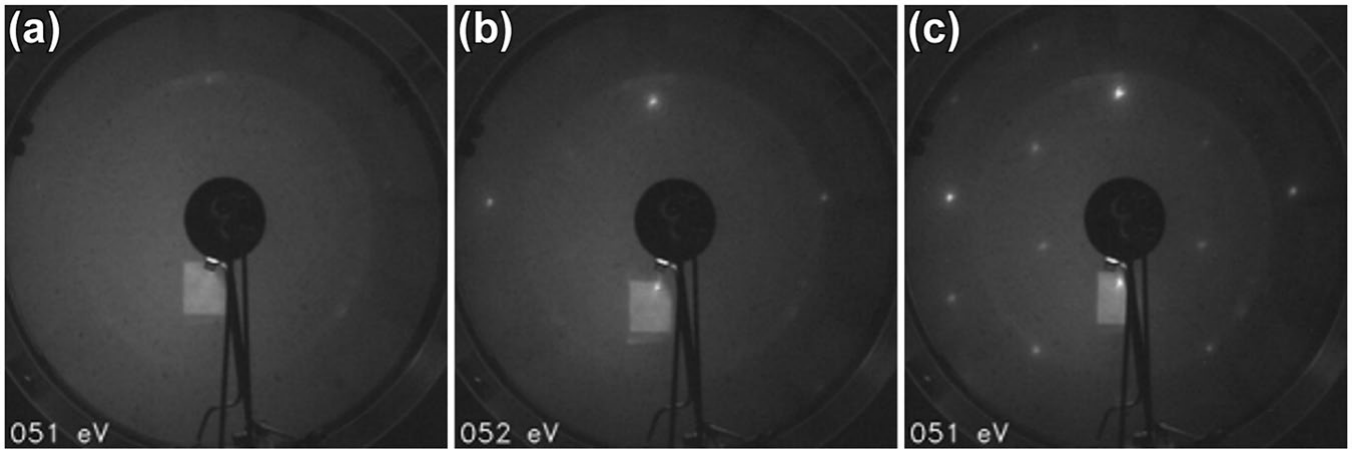
LEED images from InSb(111)B surface after (**a**) 13 500 L RT oxidation, (**b**) 13 500 L oxidation at about or slightly less than 250 °C (below pyrometer measurement range), (**c**) 13 500 L oxidation in 360 °C.

Depending on the parameters used for oxidation of clean pristine InSb(111)B(3 × 3), the resulting reconstruction is found as (3 × 3), (2 × 2), or (1 × 1) that is typically observed with simultaneous dimming of the bulk (1 × 1) spots also. With high dose oxidation treatments, a clean (3 × 3) has tendency to transform into (2 × 2). However, with too low a temperature during oxidation, the LEED spots started to blur out (Fig. 4), indicating an increasing amount of disordered oxide formation, or change from dissociative chemisorption to non-ordered, non-dissociative adsorption of O_2_ molecules.

Therefore, no direct evidence for crystalline oxide phase could be observed by diffraction, which indicates that the (111)B could be thermodynamically stable against oxidation. This is contradicted by the fact that slight lowering of the temperature seems to possibly increase disordering on the surface since the (1 × 1) LEED spots tend to blur out (for example, see Fig. 4(a,b)). Also the thermodynamics of reactions $\frac{3}{4}{{\rm{O}}}_{{\rm{2}}}+{\rm{InSb}}\to \frac{1}{2}{{\rm{In}}}_{{\rm{2}}}{{\rm{O}}}_{{\rm{3}}}+{\rm{Sb}}$ $({\rm{\Delta }}G=-\,391\,{\rm{kJ}}\,{{\rm{mol}}}^{-1})$; $\frac{3}{4}{{\rm{O}}}_{{\rm{2}}}+{\rm{InSb}}\to \frac{1}{2}{{\rm{Sb}}}_{{\rm{2}}}{{\rm{O}}}_{{\rm{3}}}+{\rm{In}}$ $({\rm{\Delta }}G=-\,292\,{\rm{kJ}}\,{{\rm{mol}}}^{-1})$; and 2 O_2_ + InSb → InSbO_4_ $({\rm{\Delta }}G=-\,806\,{\rm{kJ}}\,{{\rm{mol}}}^{-1})$ should settle the equilibrium towards the oxide phase. That is, in bulk-phase, formation of In–O and also Sb–O bonds should occur even in RT^33–35^. In this light, formation of In–O bonds is much more likely. However, conclusions based on considering bulk phases only will not suffice plainly because bonding on the semiconductor surface is markedly different than that of bulk, and above examples are for guidance in a very elemental sense. Also, an initially forming thin oxide film can passivate the material against further oxidation and thus, limiting effects of the reactions need to be considered as they proceed.

The dissociative and non-dissociative adsorption behavior of O_2_ molecules will be briefly discussed next through observations made for prototypical III–V surfaces of GaAs. Although especially the initial oxygen uptake is dependent on exposure conditions such as sources of O_2_ excitation, already early studies on oxidation of GaAs(100), (110), (111)A and (111)B surfaces have shown fairly consistent oxygen uptakes^36,37^ with initial sticking coefficients for ion bombardment and anneal (IBA) cleaned surfaces varying between these faces in the range of 3 × 10^−6^ to 3 × 10^−4^, the lowest ones reported for (110) and highest for (111)B and (100)^38^. When oxidation of GaAs(100)*c*(8 × 2) is carried out in elevated temperature, no molecular oxygen is found to desorb during Thermal Desorption Spectroscopy (TDS), characteristic at 450 °C for samples oxidized at RT^39^. Only reaction products from the surface, such as Ga_2_O and As_2_, desorb at markedly higher temperature, characteristically at ~500 °C^39,40^. For GaAs(100)*c*(8 × 2) the oxygen uptake increases dramatically with sample temperature until a saturation point, which is related to dissociative adsorption being the limiting step in oxidation^41^. The essential point to note here is that dissociative adsorption from gas phase occurs at lower sample temperatures than desorption of non-dissociatively adsorbed molecules, which is a prerequisite for controlled oxidation at elevated temperatures. Taking the effects discussed into account, we expected dissociative adsorption possibly to occur also with InSb(111)B, with the resulting surface layers still exhibiting (2 × 2) and (3 × 3) periodicities, since clear oxidation has been observed for InSb(100) with similar parameters^14,42^.

Apart from the reconstructions acquired that correspond to the ones for a clean InSb(111)B, the LEED pattern resulting from oxidation is to a certain degree also dependent on the sample preparation and cleaning procedure. We could not produce (2 × 2) on the roughened area of sample while it was readily observed on the other side after similar oxidations as for a pristine sample. Instead, rectangular lattices are observed on the roughened area, shifted by 60° rotations. Similar effect is seen for all worn out sample surfaces. More detailed discussion along with interpretation of the experimental results is presented for each of the reconstructions below.

#### (3 × 3) after oxidation

For all of the temperatures used, there was some threshold of exposure before initiation of reconstruction change from (3 × 3). Generally, higher temperatures gave a higher threshold. For example, at approximately 360 °C (3 × 3) persisted until about 3000 L to 6000 L. In this range, LEED pattern shifted to (1 × 1) along with faint (2 × 2) and to a brighter (2 × 2) with increasing exposure, but at 400 °C, only (3 × 3) was observed after at least up to 5400 L. However, on roughened samples or areas on a sample, (3 × 3) was observed throughout exposure ranges up until more than 50 000 L, alongside a rectangular semicommensurate or quasicrystalline-like lattice described later.

Using the above discussed behavior of GaAs oxidation as an analogue, the observed (3 × 3) phase after oxidation could be a result of immediate desorption of non-dissociated O_2_ molecules, leaving an adsorbant free, clean In-stabilized InSb(111)B surface due to total absence of dissociatively adsorbed oxygen. Another alternative is a novel (3 × 3)–O phase in which reconstruction periodicity is not changed with respect to the original one.

**Figure 5:**
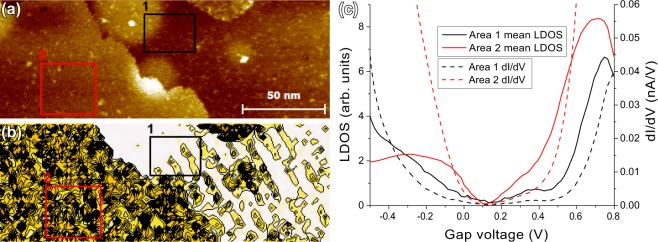
Large area images after 2000 L oxidation at 400 from (3 × 3) reconstructed surface with RT: filled state STM image (**a**), and simultaneous CITS showing I(V) contour at −0.42 V (**b**). d*I*/d*V* and *LDOS* curves (**c**) are calculated and averaged from the selected areas with corresponding colors in (**a**,**b**).

In STM, some areas exhibiting similar structure as on clean samples could be observed after these treatments. Unconventionally good resolution is necessary for observing atomic features, from e.g. (3 × 3) unit cells that encapsulate the hexamers of InSb(111)B, this is especially true for oxidized surfaces. However, here STS and CITS play an important role, giving hint about different electronic properties in different areas, even though any differences are difficult to observe with only STM. Figure 5 gives a good example about this effect for an oxidized surface after several sputterings, i.e., an already slightly worn out or roughened surface. Adjacent terrace areas separated by an atomic step may exhibit very different electronic structure, seen as higher and lower conductivity, d*I*/d*V*, which can be scaled to show proportional local density of (tunneling) states (*LDOS*) by dividing with *I*/*V*. This approach is prone to high noise at *V* = 0 where also *I* = 0, but it can be suppressed with positive offset *c* to the *I*/*V*^43^. We use

1 $$LDOS=\frac{{\rm{d}}I/{\rm{d}}V}{\sqrt{{(I/V)}^{2}+{c}^{2}}}$$

to calculate *LDOS*, in which *c* = 0.002 nA V^−1^. This approach reduces the effect of the offset on values further from *V* = 0. This way we can make more reasonable estimations of differences in band gap area due to suppressed noise but accuracy of the analysis for bands further from band gap area remain reliable.

Indeed, Fig. 5 already shows that oxidation induces a clear shift and suppression in the electronic states near the band gap. We intuitively expect the broadening of lower conductivity and *LDOS* to correspond to an oxide phase. The tunneling gap observed at roughly around 0.1 V instead of −0.1 V to 0 V is assumed to be caused by a slight offset in the STS voltage.

**Figure 6:**
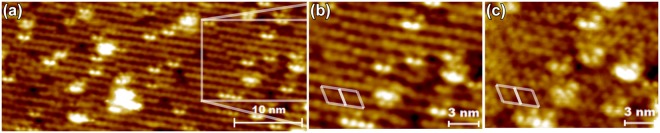
LNT STM images from (3 × 3) area after 5400 L oxidation at 400 °C: (**a**) larger and (**b**) smaller scale filled state images, and (**c**) empty state image from the same area as (**b**).

LNT STM results in Fig. 6 show atomic resolution images for a narrow area of a sample surface treated similarly, where (3 × 3) unit cells are sketched as a guide for the eye. In this case, no tilting or shifting inside any unit cell areas could be observed even during prolonged measurements lasting about 90 min. A clear dissimilarity to STM results from the clean surface in both filled, (a) and (b), and empty states (c) as well as a clear bias dependence on the observed structure distinctly show that we have indeed observed a novel InSb(111)B(3 × 3)–O phase. In filled state images (Fig. 6(a,b)), two distinct protrusions per unit cell are observed whereas in empty state images a third one is seen much more prominently below these two in Fig. 6(c). When comparing to the images of a clean pristine (3 × 3) surface regular In_4_Sb_2_ (Figs 2 and 3) which has the closest resemblance, the characteristic separate protrusions in empty state images are much more prominent and in filled state images the additional protrusion has appeared for (3 × 3)–O. Therefore, the atomic structure has clearly been modified.

#### Oxidation-induced (2 × 2)

To distinguish whether we have observed a novel InSb(111)B(2 × 2)-O phase or plainly stabilized InSb(111)B(2 × 2)-Sb (resulting from desorption of In_2_O analogously to the case of Ga_2_O in GaAs) with an oxidation treatment, we carried out annealing tests with simultaneous LEED imaging. This was done for both, (2 × 2)-Sb, prepared with Sb-deposition, and (2 × 2)-O, prepared through oxidation. It was observed that for both, (2 × 2)-Sb and (2 × 2)-O, the changes observed in the reconstructions, i.e. transition from (2 × 2) into (1 × 1) occurs at an identical stage, at approximately 380 °C to 390 °C. We find this kind of similarity of desorption behavior unlikely for structures in which the difference is between Sb–(III/V) and O–(III/V) bonds in the topmost layers. Thus, we propose that either the O atoms are able to penetrate into the subsurface of the structure, similarly as in refs^42,44,45^ [for GaSb/InSb(100)], and^46,47^ [for Si(111) and (100)], or, that oxidation results in removal of In as In_2_O molecules and concomitant stabilization of (2 × 2)-Sb.

If In_2_O desorption is the dominating mechanism and (2 × 2)-Sb a stable phase during oxidation, the observations are straightforwardly interpreted in different temperature ranges. (1) At below the temperature of Sb-desorption, i.e. 380 °C to 390 °C, oxygen atoms are inserted into the In–In bonds in each hexamer on the (3 × 3) surface, and desorb as In_2_O, leaving an Sb-rich layer which is then reconstructed accordingly to (2 × 2)-Sb. (2) At temperatures higher than this, Sb is also desorbed, revealing a (3 × 3) and exposing it to further desorption. This mechanism would thus result in a continuous net etching effect of the material. This mechanism could also explain the observation of (3 × 3) after oxidation in high temperatures, since O_2_ exposure and annealing are stopped at the same time and formation of In_2_O would cut off faster than Sb-desorption since cooling down of the sample is not as immediate as pumping out O_2_ is. On the other hand, such etching scenario would require that the substrate temperature has a key role in forming oxidation state, allowing only the low oxidation state volatile In_2_O type building blocks to form at some temperature window, because a stable (3 × 3)–O has already been observed to form at a higher temperature around 400 °C.

Thus, chemical analysis is required to distinguish the effects of O interaction on the surface. A setback in doing XPS measurements to clarify whether an oxide is formed, is that the analysis is integrated over a large (approximately 1 mm^2^) area. This means that signal is averaged also over defective areas, which are always more or less present on the surface. Such areas that could contain, e.g., In-droplets could oxidize much more readily^48^ in some specific conditions and cause misinterpretations from the analyzed signal. To avoid such problems, we performed XPS for (i) cleaned InSb(111)B oxidized in room temperature (RT) at 5 × 10^−6^ mbar for 60 min and 120 min (two subsequent 60 min exposures), (ii) similarly for cleaned InSb(111)B oxidized at about 370 °C. LEED pattern for the treatments corresponded to faint (1 × 1) and (2 × 2) as in Fig. 4(a,c), respectively. The faint (1 × 1) is a convincing evidence of the adsorption of oxygen on a monolayer range, which is a good reference point for comparison based on XPS results.

**Figure 7:**
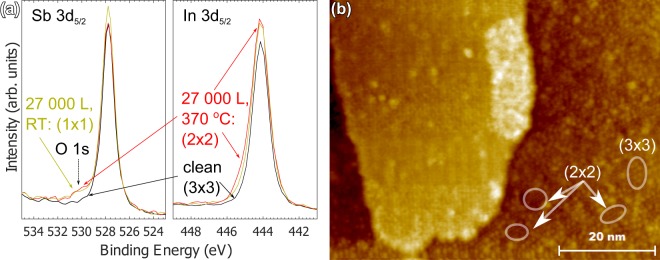
(**a**) XPS results from clean (3 × 3), RT oxidized (1 × 1) and 370 °C oxidized (2 × 2). Intensity ratio between Sb 3d_5/2_ and In 3d_5/2_ is seen to grow after oxidation in elevated temperature as well as O 1 s intensity similarly as in RT. The systematically higher intensity in RT oxidized sample is expected to come from possible variation in x-ray gun positioning but does not affect the analysis. (**b**) STM image from partly (2 × 2) containing area after oxidation: after 2250 L at 350 °C, *I*_*t*_ = 53 pA, *V*_*g*_ = 1.22 V, with residual (3 × 3) structure and oxidation induced growth of island structure not observed on clean (2 × 2)-Sb.

Figure 7(a) shows comparison of the XPS spectra between clean, RT oxidized and 370 °C oxidized samples (5 × 10^−6^ mbar, 120 min). We firstly note that O 1 s signal is observed from XPS data for both of the oxidized surfaces. Taking into account the observation of faint (1 × 1) still indicating oxygen adsorption on the clean surface, we interpret that the oxygen content is in a monolayer range. However, oxidation-induced (2 × 2) reconstructed surface contains similar or even slightly higher amount of oxygen, with subtle but clearly visible core-level shifts and broadening in In 3d, and increased FWHM also in Sb 3d. The results indicate distinct bonding of oxygen onto the semiconductor structure in the (2 × 2) areas since emission from only defective sites would be expected to result in a significantly lower O 1 s signal than in the case of (1 × 1). Only rough peak fitting was carried out due to limited resolution of the analyzer, and also due to the fact that qualitative differences are distinct with naked eye. It was observed from the 3d peaks that the In:Sb ratio slightly increases from (3 × 3) after oxidation to (2 × 2), probably due to desorbed Sb, but decreases after Sb deposition and stabilization of (2 × 2)-Sb (not shown), as expected. To compare atomic concentrations in the volume limited by XPS probing depth, we used the integrated peak areas scaled with their respective atomic sensitivity factors (ASFs). From the rough analysis, we estimate the amount of oxygen in the measured signal be in the range of O/(In + Sb + O) = 10%. Assuming a roughly 0.5 nm monolayer thickness and bonding of oxygen atoms only in topmost layers, the O coverage is estimated to be about 0.25 ML to 0.5 ML. The XPS results give significant support that we have produced a novel (2 × 2)-O surface through insertion of O in the structure, most probably stabilized by In–O bonds and desorbed Sb.

XPS analysis gives good support for the chemical interaction analysis especially due to difficulty in making any distinctions of atomic configuration or density of states in STM imaging of the (2 × 2). STM image containing both (3 × 3) and (2 × 2) after oxidation is shown in Fig. 7(b). For (2 × 2)–Sb surface, similar features are observed everywhere in large scale, but for (2 × 2)–O with parameters on the lower boundary of (3 × 3) → (2 × 2) transformation (2250 L at 350 °C), we observe individual (3 × 3) and (2 × 2) unit cells along with islands with markedly different periodicity. The islands are expected to originate from residual In or Sb nucleated as islands, which is supported by STS measurements showing more or less metallic character of these areas (non-negligible *LDOS* at 0 V, not shown). STS comparison between clean (3 × 3) or (2 × 2)–Sb and (2 × 2)–O has not been done as they have not been observed reliably in any adjacent areas, which would be necessary for separating such subtle differences.

We note that the resulting (2 × 2) remains in well-ordered crystalline structure even after exposures as high as 130 000 L at 370 °C, which indicates a clearly passivating effect. This promotes the utilization of the thermal oxidation treatment beneficial in further processing of potential III–V/oxide device structures.

#### Incommensurate oxide growth

**Figure 8:**
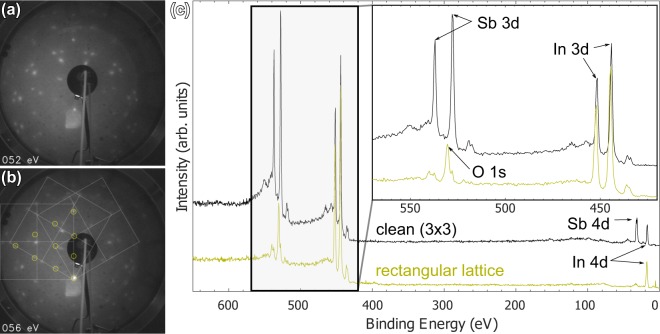
LEED images from roughened area after 50 000 L oxidation at about 390 °C at (**a**) 52 eV and (**b**) 56 eV with (3 × 3) spots indicated with yellow and $(\frac{3}{2}\times \frac{3}{2})$-sq squares sketched. (**c**) XPS survey spectra from clean (3 × 3) and heavily oxidized rectangular lattice surface transferred to XPS system through air. Spectrum baselines have been offset for clarity. Zoomed-in inset from Sb 3d + In 3d range is also shown. Enrichment of In along with Sb deficiency is prominent in rectangular lattice structure.

A prolonged oxidation in similar conditions stabilizes and enhances a peculiar lattice which matches the substrate only in one direction, i.e., a semicommensurate rectangular phase on a roughened sample. The diffraction pattern is seen in Fig. 8(a). A few squares corresponding to a superstructure lattice constant of $\frac{3}{2}$ × bulk lattice constant along with yellow circles indicating (3 × 3) spots are also sketched in Fig. 8(b). This square unit cell, henceforth denoted $(\frac{3}{2}\times \frac{3}{2})$-sq with three domains of 60° rotation is seen to reproduce the LEED pattern fairly well apart from residual (3 × 3) pattern and spots adjacent to (3 × 3) in (1 × 1) lines of the substrate unit cell.

The stability of this oxide phase was tested with similar annealing as for (2 × 2)–O while monitoring LEED. The (3 × 3) spots were observed to disappear roughly at 250 °C while the superstructure spots remained bright even when annealing to over 400 °C. In addition, the sample was transferred in the atmosphere to the other UHV system to check elemental composition with XPS. After annealing to 370 °C to remove adsorbents from the air, the square lattice spots reappeared. This suggests a highly stable nature of the oxide. The XPS results in Fig. 8(c) show that the film mainly consists of In oxide, with In:Sb ratio being more than 5:1, when taking atomic sensitivity factors (ASFs) of the 3d peaks into consideration^49^. It is possible that the rectangular lattice film consists entirely of In oxide, since Sb oxide signal could originate from areas that were not well covered in the initial oxidation treatment and thus, oxidized in air. After annealing to remove atmospheric adsorbents, substrate bulk Sb 3d_5/2_ signal is also visible, indicating that the film thickness is in the range of few nanometers.

**Figure 9:**
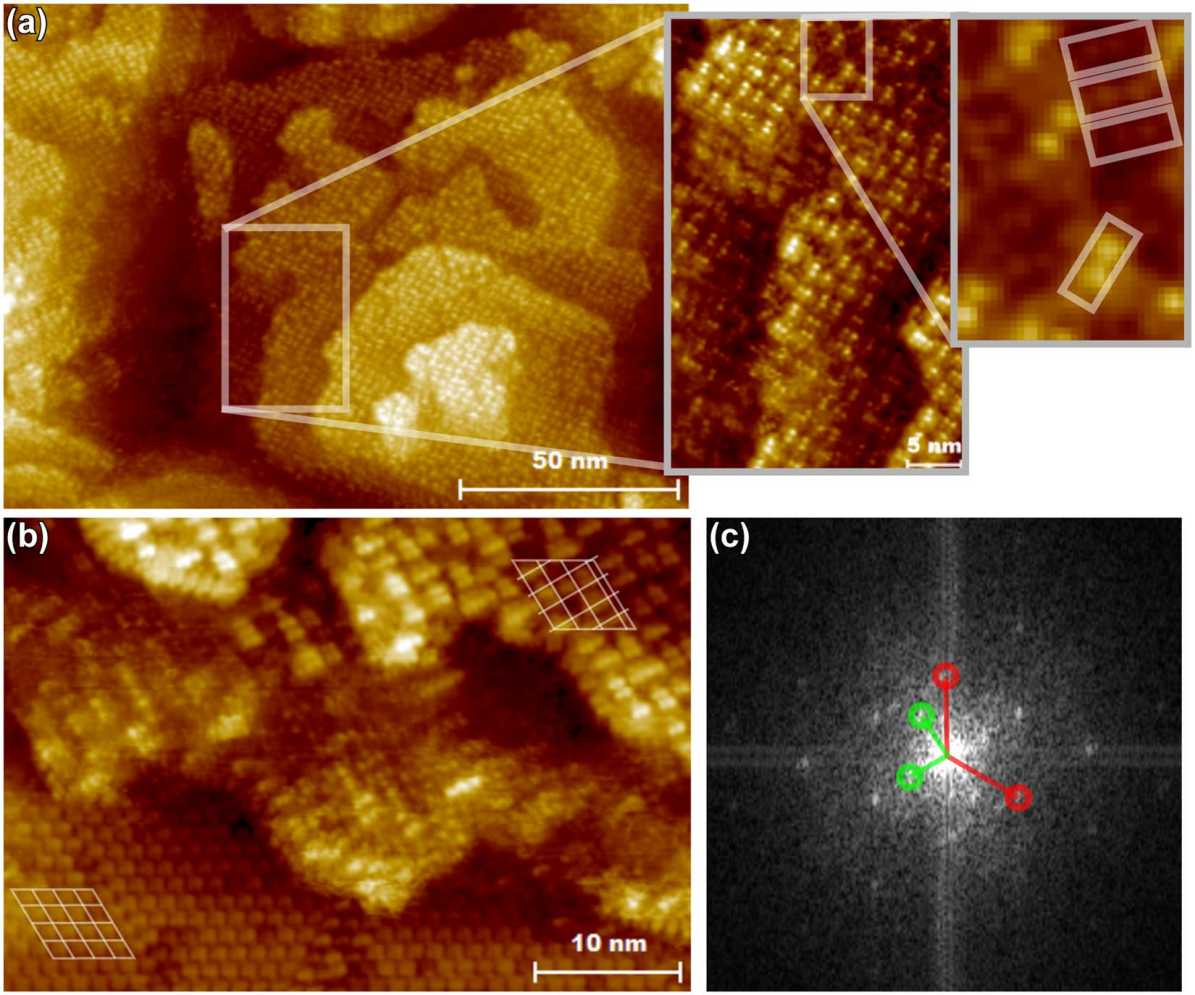
STM images from roughened area after 50 000 L oxidation at about 390 °C: Large scale area with multiple rectangular lattice featured islands along with zoomed-in areas to distinguish atomic features (**a**). Area with semicommensurate quadratic reconstruction and hexagonal (3 × 3) (**b**). Fast fourier transformation (FFT) image from (**b**) with both axis spanning 2.45 nm^−1^ (**c**). *I*_*t*_ = 100 pA, *V*_*g*_ = 2.00 V.

Additional information from the structure discussed above can be obtained from STM measurements such as in Fig. 9(a), which shows that the lateral structure is also somewhat complex and could account for the additional LEED spots. It is observed that the superlattice has no long range periodicity on the sample scale and adjacent islands are often rotated 30° or 60° with respect to each other. In fact, dimer-like features observed on the topmost atomic layer have a 45° rotated orientation on some areas of the 2nd layer. On the other hand, even the orientations of these dimer-like features are seemingly random in steps of 90° on the topmost layer. It is observed in the zoomed-in areas of Fig. 9(a) that the random orientation of dimer-like units breaks translational symmetry, so that periodicity is not maintained, but the possibilities for local short-range ordering are limited, so that ordering is. It is not apparent whether these effects are mostly dictated by the substrate, growth mode, or local defects. However, it is noted that on average, for the dimers that have 3 or 4 dimers as nearest neighbors in Fig. 9(a) zoom-in, about 70% of the neighbouring dimers are in perpendicular orientation, seemingly favoring this positioning, and perfect tiling of such units would produce a perfect square lattice. In the 2D structure, dimensional separations of adjacent parallel and perpendicular dimers are different so that even one exception from this rule causes significant deformation, multiplying in other units and this causes the long-range translational periodicity to vanish, producing the quasicrystal-like structure.

Figure 9(b) shows an STM image with local (3 × 3) structure next to a rectangular lattice island. The features in (3 × 3) structure closely resemble the hexamers in parallel orientation as in Fig. 2(c–f), but there is also an area in the bottom middle part of the image where similar structure is seen as in (3 × 3)–O of Fig. 6. Having these areas visible simultaneously is useful for calibration of the length scale. Also, it is of particular interest that we have observed the hexamers of the clean surface here in parallel orientation in RT. We used fast fourier transformation (FFT) in Fig. 9(c) from the area in (b) to scale the (3 × 3) unit cell (red) dimensions to 1.37 nm × 1.37 nm with an angle of 120°. Subsequently, the real space unit cell dimensions of the nearest quadratic spots (green) were measured (2.14 nm × 2.14 nm, 90°) and used in the calibration of the dimensions of the following images, since they were visible in all of the FFT’s of the STM images from rectangular lattice areas.

**Figure 10:**
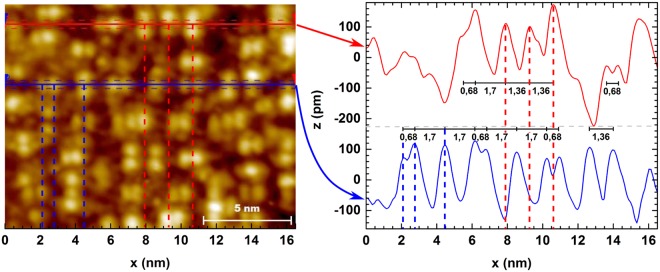
A narrow scale image from area with a rectangular lattice as in Fig. 9 with a higher resolution along with height curves averaged over the area limited by the horizontal dashed lines. Distances between the most common features are indicated in the right handlower pane height curve diagram.

Using this calibration and scaling an area with a higher attained resolution seen in Fig. 10 we observe that the average spacings of individual protrusions are indeed approximately 0.68 nm, corresponding to the $\frac{3}{2}$ × bulk lattice constant, and corresponds roughly to a lattice constant of this square-superstructure. The other axis of this rectangular superstructure is always in parallel direction with one of the lattice vectors of the surface (3 × 3) unit cells but the other one, being perpendicular, makes the lattice semicommensurate without the $n\times \sqrt{3}m$ periodicity required for matching a hexagonal lattice (see e.g. upper right corner of Fig. 9(b)).

From Fig. 10, one can derive a multiplicity of local unit cells describing the short range ordering on this structure, the dominant ones in rectangular coordinates having spacings indicated in the right hand pane. However, due to the somewhat randomly oriented dimer and trimer like units, oblique and other non-conventional periodicities are prominent in most areas. This explains some of the complexity in the LEED patterns and also some of the features observable in STM FFT, but not LEED.

To give justified suggestions for the stages of growth for this square lattice, we summarize the observations related to the roughened areas where and only where $(\frac{3}{2}\times \frac{3}{2})$-sq grows: (**1)** (2 × 2) is not observed in these areas. (**2)** (3 × 3) hexamers tend to set in parallel orientation. (**3)** Existence of (3 × 3)–O has been verified. (**4)** (3 × 3) and (3 × 3)–O are observed alongside $(\frac{3}{2}\times \frac{3}{2})$-sq after oxidations. (**5)** Growth of $(\frac{3}{2}\times \frac{3}{2})$-sq at least laterally is much slower than (2 × 2)–O. (**6)** $(\frac{3}{2}\times \frac{3}{2})$-sq is the smallest unit cell of this structure, but other periodicities also exist. (**7)** The rectangular lattice oxide consists mainly or entirely of In oxide.

It is therefore clear that $(\frac{3}{2}\times \frac{3}{2})$-sq grows on areas where parallel (3 × 3) and consequently also (3 × 3)–O is initially observed. We suggest that the most stable oxide phase is achieved by inserting O into In-bonds which results in (3 × 3)–O when the hexamers are initially stabilized in parallel orientation, which seems to be the case in roughened areas or such that are rich in step-edges. As the oxidation proceeds further from the (3 × 3)–O phase, strain is likely induced into the surface layers, which will decompose the hexagonal surface structure after some threshold, driven by the tendency of In to form oxide bonds preferentially over Sb. A semicommensurate lattice is formed, which indicates some degree of relaxed bonding to the substrate, assisted by the ability of the film constituents to relax in multiple orientations without making the structure amorphous. In fact, the observation of solid-state quasicrystal phase has been reported for metal–metal and metal–oxide systems^50^, but not for semiconductor–oxides. Therefore, the observations and analyses presented here pave way for new aspects on research of semiconductor–oxide interfaces.

The rectangular lattice oxide described here is distinctly a multi-layered structure, which is why the density of states or defect levels at the interface cannot be directly probed with STS. This would require device tests or valence band data, which are beyond the scope of this investigation. Provided that defect level density follows the same trend as in the case of (3 × 3) → (3 × 3)–O, which seems to be the initial stage of growth, we note that growth of this oxide could be highly beneficial for device interfaces due to its significant thermal stability. The observation of this rectangular lattice phase in areas with high density of step edges and our other results above indicate that similar phases could form spontaneously during similar oxidations for InSb-based 3D device structures. In such structures feature sizes are in the nanometer length scale, where the parallel (3 × 3) unit cell structure is likely stabilized due to vicinity of step edges, which will in turn stabilize the $(\frac{3}{2}\times \frac{3}{2})$-sq growth.

## Conclusions

In summary, we have observed crystalline phases on InSb(111)B induced by thermal oxidation. Interesting features characterize these surfaces, namely, they reconstruct either with identical periodicity as the clean surface, or with semicommensurate phases. The evidence provided by STM, STS and XPS indicate that even the phases reconstructed identically as the clean surface do have oxygen-induced characteristics and thus are categorized as crystalline oxide phases, that have now been discovered for the first time for crystal planes other than (100). The mechanisms behind transformation from InSb(111)B(3 × 3) to (3 × 3)–O, (2 × 2)–O and $(\frac{3}{2}\times \frac{3}{2})$–sq have been discussed, and the analysis brings forth new possibilities for viable semiconductor–oxide interface structures. Furthermore, the formation of these phases can be carried out after simple cleaning method of chemical native oxide removal and annealing. The findings and results are of major significance concerning the utilization of crystalline oxide passivation for non-planar III–V/oxide devices, especially concerning the fact that the treatment is transferable to device manufacturing process flow.

## Methods

### Samples and equipment

5 × 10 mm^2^ sample pieces were cut from *n*-InSb(111)B (±0.01°, Te-doped, 1.1 × 10^15^ cm^−3^ to 2.3 × 10^15^ cm^−3^) wafer and transferred on an Omicron flag style sample holder to an ultra high vacuum (UHV). The investigations were carried out in two separate UHV systems. Both of the systems contain a preparation chamber equipped with a radiative sample heating tungsten filament and O_2_ leak valve with a base pressure in the range of 10^−9^ mbar. Both of the systems also have low energy electron diffraction (LEED) equipment, and a commercial Omicron SPM chamber with sample heating, Ar^+^ sputtering and STM equipment. The base pressure is in low 10^−10^ mbar range in SPM chamber during measurement. There is also x-ray photoelectron spectroscopy (XPS) equipment in the analysis chamber of one of the systems, which was used for chemical identification and analysis for the samples after treatments. Sample temperatures were measured with an IR pyrometer with a measurement range of 250 °C to 600 °C. We anticipate a maximum error margin of ±20 °C for the measured temperatures.

### Surface cleaning

Samples were cleaned in UHV by cycles of simultaneous Ar^+^-sputtering (0.8 kV to 1 kV, 15 min to 20 min) and annealing at 250 °C to 300 °C followed by post-annealing at 300 °C to 400 °C (30 min). In order to study how the controlled pre-oxidation procedure could be incorporated into an industrial process flow, some InSb subtrates were cleaned by HCl-based etching of native oxide instead of Ar^+^-sputtering. HCl:IPA (1:3) etching for 150 s combined with IPA rinse for 60 s was used before the sample transfer via air (about 1 air exposure) to vacuum system. Separate samples were annealed in UHV system with and without 5 × 10^−5^ mbar H_2_ ambient for 30 min first at 300 °C and then at 360 °C to remove atmospheric impurities and investigate whether atomically clean and smooth surface could be obtained.

### Reconstruction modification

To compare the properties of reconstructions produced by different methods, smooth Sb-rich InSb(111)B(2 × 2) was produced by depositing Sb by means of thermal evaporation onto sputter cleaned starting surface, while keeping the sample at about 380 °C. In this temperature, excess metallic Sb is expected to desorb, so that the exact amount of Sb deposited is not of high relevance. To observe and utilize the (3 × 1) phase, we also tested annealing the sample at UHV environment to about 470 °C (for approximately 30 min) as in ref.^23^.

### Oxidations

The samples with a clean surface were oxidized with an O_2_ pressure of 1 × 10^−6^ mbar to 2 × 10^−5^ mbar for 15 min to 60 min while keeping the sample temperature at 350 °C to 400 °C. Oxidations were tested particularly in combinations that closely correspond to InSb(100)(3 × 1)-O and InSb(100)(1 × 2)-O oxidation conditions.

### LEED

Measurements were carried out for each experiment after cleaning the sample and oxidation. Typical beam energy was 50 eV to 60 eV and screen voltage 5 kV.

### LEED with simultaneous annealing

Heating filament current was increased step by step in 5 min intervals and sample heating stage temperature was monitored simultaneously. Relation between sample stage and sample temperatures was calibrated with a pyrometer separately, and the corresponding sample temperatures were checked for each stage.

### STM

Room temperature (RT) STM and STS measurements were carried out to investigate the exact atomic orientations and their possible differences between clean and oxidized surfaces with representative treatments: cleaned (3 × 3) and oxidized for each of the reconstructions observed. For a clean as well as low dose oxidation experiment, to reduce noise occurring from thermal effects and this way improve stability and resolution also liquid nitrogen temperature (LNT) STM characterization was done. LNT STM and STS were performed under LN_2_ cooling at about 85 K with maximum temperature drift at STM scanner being 2 mK min^−1^. All of the STM measurements were carried out in constant-current mode, using a tunneling current *I*_*t*_ = 100 pA and a gap voltage *V*_*g*_ = 1.00 V (negative for filled and positive for empty states) unless otherwise stated. Omicron STM-1 and Fermi SPM systems were used for STM characterization.

### XPS

Measurements were done using a home laboratory system with non-monochromatized Mg K*α* x-ray source and a hemispherical electron energy analyzer. All of the spectra were taken with normal emission. Survey spectra for checking the general composition on the surface were measured with 30 eV pass energy. Pass energy of 15 eV was used for measuring In 3d and Sb 3d with partly overlapping O 1 s binding energy (BE) ranges with 0.2 eV step size. For the In 3d and Sb 3d where main contribution is expected to come from bulk emission, the peak maxima are aligned to the same BE to suppress possible variation in spectrometer work function between measurements and in this way distinguish the subtle features more reliably. Savitzky-Golay quadratic smoothing algorithm was used with a window size of 5 data points to emphasize the systematic differences between spectra. Background levels were scaled before and after the main peak linearly to the same level for similar spectra, in order to equalize the baseline between different measurements and thus enable reliable comparison of intensity magnitudes.

### Simulations

The atomic structure for clean (3 × 3) surface^20^ was simulated computationally, and was utilized for STM simulations to compare to our measured data. Calculations were performed by using Vienna ab initio simulation package (VASP)^51–54^, applying the projector augmented wave (PAW) method^55,56^ and the Perdew-Burke-Ernzerhof (PBE) generalized gradient approximation^57^. The atomic structure was optimized by using conjugate-gradient minimization of the total energy with respect to the atomic coordinates. The plane wave cutoff was 350 eV and the *k* mesh of the (111)B(3 × 3) surface unit cell was 3 × 3 × 1. The constant current STM images were simulated within the Tersoff-Hamann approximation^58,59^.

## Data Availability

All relevant data generated or analysed during this study are included in this published article. Raw data for the XPS spectra and STM/STS figures generated during and analysed during the current study are available from the corresponding author on reasonable request.
